# 

**Published:** 1908-08-15

**Authors:** 


### ANNOUNCEMENTS.

MISSOURI STATE DENTAL ASSOCIATION.—At the 43rd annual meeting of the Missouri State Dental Association, held at the Planter's Hotel, St, Louis, the following officers were elected for the ensuing year: J. B. McBride, Springfield, President; R. E. Darby, Springfield, 1st Vice-President; E.

P. Dameron, St. Louis, 2nd Vice-President; H. H. Sullivan, Kansas City, Recording Secretary; J. F. Wallace, Canton, Corresponding Secretary; J. T. Fry, Moberly, Treasurer. Meeting of 1909 to be held at Kansas City.

ILLINOIS STATE DENTAL SOCIETY.—At the forty-fifth annual meeting of the Illinois State Dental Society, the following officers were elected for 1908–1909: President, Arthur D. Black, 31 Washington St., Chicago; Vice-President, E. F. Hazell, Springfield; Secretary, R. J. Hood, Sparta; Treasurer, C. P. Pruyn, 92 State St., Chicago; Librarian, J. T. Cummins, Metropolis City. The 1909 meeting of the Society will be held at Danville, May 11, 12, 13, 14.

NOTICE FROM N. D. A. COMMITTEE ON THE HISTORY OF DENTISTRY.—Dr. Guerini's History of Dentistry.—Soon after the Buffalo meeting of the National Dental Association in 1905, this committee announced, through the dental journals and otherwise, the exceptional opportunity which has made it possible to place before the dental profession a comprehensive history of dentistry from the earliest times down to the early days of the nineteenth century. Dr. Vincenzo Guerini of Naples, Italy, well known as a dental historian and archeologist, has placed at the disposition of the committee the result of his labors in the field of dental history, which has formed a large and important part of his life work.

On careful estimate, the price of subscription was placed at five dollars per copy, and it was found that not less than 700 copies would have to be subscribed for in advance, in order to guarantee the cost of publication.

The matter is ready to put in type, and the book can and will be rapidly put through the press just as soon as the amount necessary to accomplish that end is available for use by the committee.

Send your subscription without further delay to Dr. Chas. S. Butler, treasurer, "The Frontenac," Buffalo, N. Y. Speak of the matter favorably to your colleagues and induce them to do likewise, so that this much-desired object may be consummated without any undue delay.

Edward C. Kirk, Philadelphia.
Wm. H. Trueman, Philadelphia.
Gordon White, Nashville, Tenn.
H. L. Ambler, Cleveland, Ohio.
Jas. McManus, Hartford, Conn.
J. Y. Crawford, Nashville, Tenn.
A. H. Fuller, St. Louis, Mo.
S. A. Freeman, Buffalo, N. Y.
W. E. Boardman, Boston, Mass.
Charles S. Butler, Buffalo, N. Y.

Burton Lee Thorpe, *Sec'y*, St. Louis, Mo.

Chas. McManus, *Chairman*, Hartford, Conn.

---

A New Edition of Gray's Anatomy.—As a matter of both importance and interest to our readers the announcement of a new edition of GRAY'S ANATOMY stands in the forefront of literary events. A thorough revision has been in progress for about two years and is so near completion that publication will be made in about a month.

---

Dental Manufacturers' Exhibit.—Arrangements have been completed for the next Dental Manufacturers' Exhibit to be held at the Sinton Hotel, Cincinnati, Ohio, on October 27, 28, 29 and 30.

Cincinnati is selected for this exhibit for the purpose of giving the dentists in the central states, who could not attend the exhibition held in March, in New York, an opportunity to attend this one. The interest shown by the profession at that time seems to justify a continuance of these exhibits in other parts of the country.

The product of the leading Dental Manufacturers of the United States, is fully demonstrated at these exhibitions, among them being the latest inventions in all lines of dentistry and they are not only interesting but educational.

No expense is spared to make these exhibitions a success and the location of the October exhibition is exceptionally good, being in the best hotel in the city.

Special arrangements have been made for the comfort and entertainment of the lady visitors, and the four days spent in the city can be filled in a most beneficial and interesting manner.

There is no fee of admission, buttons being furnished on application at the registration desk, admitting the wearer to the exhibition hall and clinics at all times.

All dentists and their families are invited to attend and special hotel arrangements are being made for the out of town guests.

A. C. CLARK, *President.*

---

NEW YORK, N. Y., June 12, 1908.

Editor of THE DENTAL REGISTER:

Dear Sir:—I take pleasure in informing you that at the recent national meeting of the American Medical Association just held in Chicago, it was my pleasure in the House of Delegates to introduce the following resolutions:

"Whereas, The value of the services of the dental corps of the army is now thoroughly recognized,

"Resolved, That the legislative committee be instructed to further such legislation as will place the dental corps in the army on a commissioned basis, such legislation to meet the approval of the War Department."

They were referred to the reference committee on legislation, who brought in the following report, which was unanimously passed by the House of Delegates:

"The House of Delegates last year endorsed a bill, providing for the dental corps of the navy, and it seemed desirable to make the proposed resolution more general so as to embrace both branches of the science, and the committee recommends the adoption of the following resolutions:

"The House of Delegates of the American Medical Association recognizing the great importance of the services of the dental corps of both navy and army, and appreciating the importance of placing both on a commissioned basis, authorizes the committee on legislation to assist in securing the passage of such bills as meet the approval of the War Department, or the chief of the Bureau of Medicine and Surgery of the Navy."

The work was made much easier by the magnificent amount of missionary work done by the dentists throughout the country, especially in the middle and far west and southwest, as I found that the delegates from these sections appeared to be thoroughly posted by their local dentists as to the matter at issue. This, in no light measure, contributed to the result attained, which I am confident will lead to the placing of the army corps on a commissioned basis this coming winter. I have the personal assurance that the committee on legislation of the American Medical Association will use the same efforts to obtain successful legislation in this matter as they have used in obtaining the reorganization of the medical department of the army.

Respectfully yours,

M. L. RHEIN,

Delegate from the Stomatological Section.

